# Polar microalgae extracts protect human HaCaT keratinocytes from damaging stimuli and ameliorate psoriatic skin inflammation in mice

**DOI:** 10.1186/s40659-023-00454-1

**Published:** 2023-07-13

**Authors:** YoonHee Lim, So-Hyun Park, Eun Jae Kim, HeeJun Lim, Jinsun Jang, In-Sun Hong, Sanghee Kim, YunJae Jung

**Affiliations:** 1 Department of Microbiology, College of Medicine, Gachon University, 155 Gaetbeol-ro, Yeonsu-gu, Incheon, 21999 Korea; 2 Lee Gil Ya Cancer and Diabetes Institute, Gachon University, Incheon, 21999 Korea; 3 Department of Health Science and Technology, Gachon Advanced Institute for Health Science & Technology, Gachon University, Incheon, 21999 Korea; 4 Division of Life Sciences, Korea Polar Research Institute, Incheon, 21990 Korea; 5 Department of Food Science and Biotechnology, Gachon University, Seongnam, 13120 Korea; 6 Department of Molecular Medicine, College of Medicine, Gachon University, Incheon, 21999 Korea

**Keywords:** Polar microalgae, Compound profiles, Barrier protection, Psoriasis, Anti-inflammation

## Abstract

**Background:**

Polar microalgae contain unique compounds that enable them to adapt to extreme environments. As the skin barrier is our first line of defense against external threats, polar microalgae extracts may possess restorative properties for damaged skin, but the potential of microalgae extracts as skin protective agents remains unknown.

**Purpose:**

This study aimed to analyze compound profiles from polar microalgae extracts, evaluate their potential as skin epithelial protective agents, and examine the underlying mechanisms.

**Methods:**

Six different polar microalgae, *Micractinium sp*. (KSF0015 and KSF0041), *Chlamydomonas sp*. (KNM0029C, KSF0037, and KSF0134), and *Chlorococcum sp.* (KSF0003), were collected from the Antarctic or Arctic regions. Compound profiles of polar and non-polar microalgae extracts were analyzed using gas chromatography-mass spectrometry (GC-MS). The protective activities of polar microalgae extracts on human keratinocyte cell lines against oxidative stress, radiation, and psoriatic cytokine exposure were assessed. The potential anti-inflammatory mechanisms mediated by KSF0041, a polar microalga with protective properties against oxidative stress, ultraviolet (UV) B, and an inflammatory cytokine cocktail, were investigated using RNA-sequencing analysis. To evaluate the therapeutic activity of KSF0041, an imiquimod-induced murine model of psoriatic dermatitis was used.

**Results:**

Polar microalgae contain components comparable to those of their non-polar counterparts, but also showed distinct differences, particularly in fatty acid composition. Polar microalgae extracts had a greater ability to scavenge free radicals than did non-polar microalgae and enhanced the viability of HaCaT cells, a human keratinocyte cell line, following exposure to UVB radiation or psoriatic cytokines. These extracts also reduced barrier integrity damage and decreased mRNA levels of inflammatory cytokines in psoriatic HaCaT cells. Treatment with KSF0041 extract altered the transcriptome of psoriatic HaCaT cells toward a more normal state. Furthermore, KSF0041 extract had a therapeutic effect in a mouse model of psoriasis.

**Conclusions:**

Bioactive compounds from polar microalgae extracts could provide novel therapeutics for damaged and/or inflamed skin.

**Supplementary Information:**

The online version contains supplementary material available at 10.1186/s40659-023-00454-1.

## Background

The skin is a crucial organ that protects internal organs and provides an interface between the body and the environment [1]. The epidermis, the outermost skin layer, protects against external dangers such as ultraviolet (UV) radiation, injury, and pathogens [1, 2], and is comprised of keratinocytes, which make up 90–95% of epithelial cells, melanocytes, and rare Merkel cells [1, 3]. Keratinocytes and melanocytes protect the skin against UV radiation-induced oxidative stress and DNA damage by regenerating damaged cells and filtering UV radiation [4, 5]. The epithelium also harbors resident immune cells, including dendritic epidermal T cells and Langerhans cells, that coordinate homeostasis and inflammatory responses [6]. Dysregulation of keratinocyte functions can lead to various inflammatory skin disorders, such as atopic dermatitis and psoriasis, by triggering an inflammatory cascade of immune cells in the skin [1]. The skin epidermis is not merely a physical barrier against the external environment but also an active part of the immune response, playing a critical role in maintaining immune homeostasis. However, current strategies for protecting or repairing the epithelial barrier are limited.

The polar regions, with a significant proportion of Earth’s biomass in low-temperature environments, represent a largely untouched biological resource [7]. To sustain growth at low temperatures, polar organisms acquire metabolic, structural, and functional adaptations [8]. Thus, natural products synthesized by polar organisms may offer advantages for protecting against environmental challenges [9]. Microalgae are photosynthetic eukaryotes that make up a significant portion of marine and freshwater phytoplankton and have a long history of evolutionary and adaptive diversification to various habitats and extreme polar environmental gradients [7, 10]. This success in environmental adaptation makes polar microalgae ideal candidates for drug discovery, as they may have unique compounds for defense and survival not present in non-polar microalgae.

Previous reports have described the antioxidant, anti-inflammatory, and anticancer potential of polar microalgae and the secondary metabolites of polar lichens, including ramalin and lobaric acid, against human cervical adenocarcinoma or colon carcinoma cells [11–14]. Furthermore, extracts from macroalgae in the Antarctic region have demonstrated antifungal and anticancer effects against a human squamous cell line, with minimal or no cytotoxicity against normal cell lines [15]. Recent advancements in high-throughput molecular techniques have unveiled the structural components of polar microalgae that hold potential for biotechnological applications [7]. These structural components, as well as secondary metabolites, may offer advantages in protecting against environmental challenges. While the lipid plasticity of polar microalgae has recently been applied to biotechnology [16], their potential as pharmaceuticals, especially in protecting exposed epithelial tissues against damaging environmental stimuli, remains largely unknown.

We collected several species of microalgae from the polar regions, including *Micractinium sp*. (KSF0015 and KSF0041), *Chlamydomonas sp*. (KNM0029C, KSF0037, and KSF0134), and *Chlorococcum sp*. (KSF0003). The composition of these polar microalgae was analyzed and compared to that of non-polar microalgae of the same genus. We evaluated the biological activity of polar microalgae extracts in protecting against damage induced by oxidative stress and UVB in human HaCaT keratinocyte cells. We determined the anti-inflammatory activity of polar microalgae extracts in a psoriatic keratinocyte model by assessing viability, permeability, and transcriptional profiles. Furthermore, we evaluated the therapeutic efficacy of topical application of extract from KSF0041, a polar microalga that protected HaCaT cells from oxidative stress, UVB, and inflammatory cytokine stimuli, in a murine model of psoriatic inflammation. Using these assays, we aimed to evaluate the protective potential of polar microalgae extracts in damaged and inflamed skin and examined the underlying mechanisms.

## Results

### GC-MS profiling of microalgae extracts

**Fig. 1:**
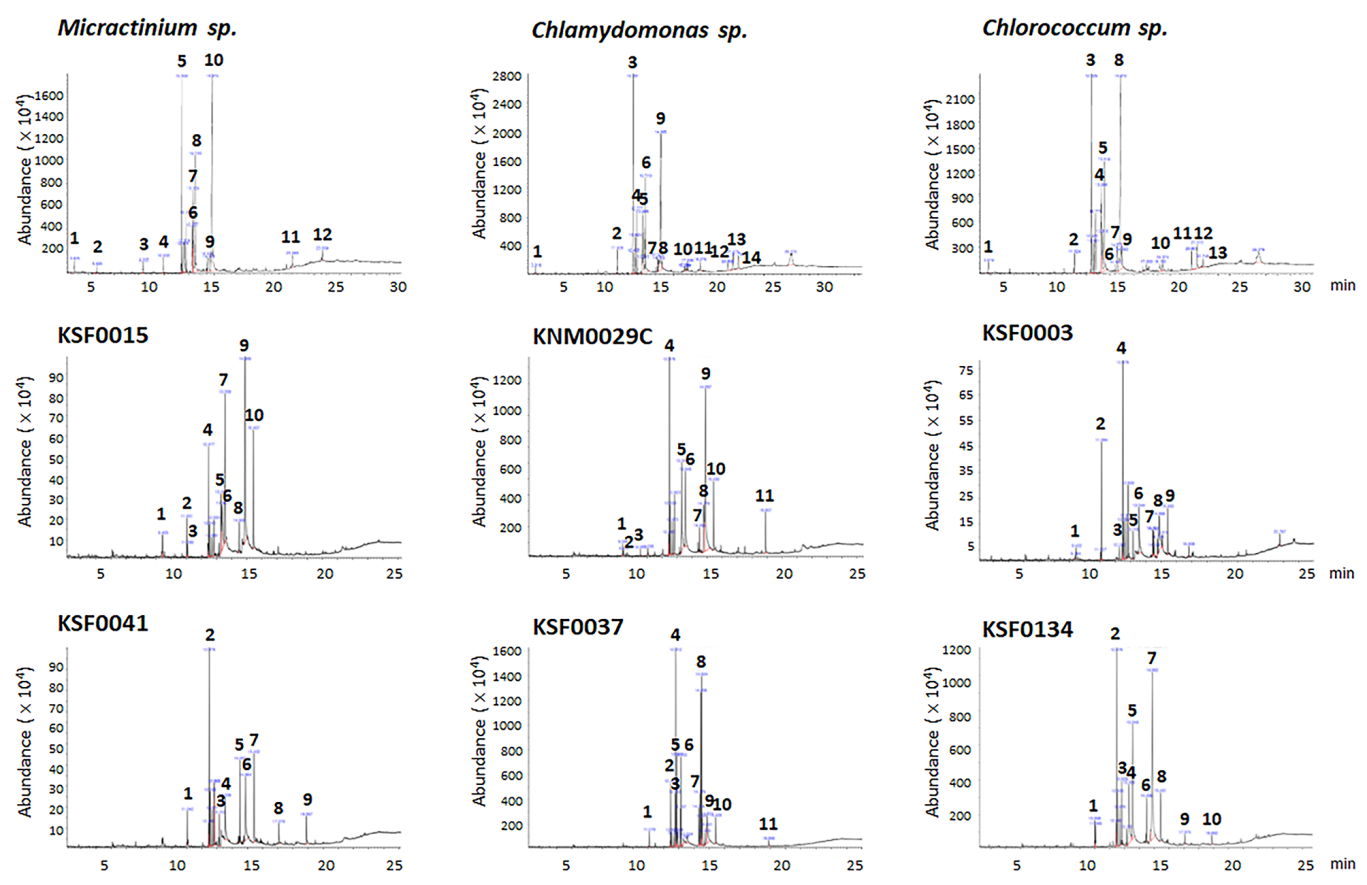
Chromatographic profiles of the non-polar and polar microalgae extracts. The corresponding compound identities are listed in Tables 1, 2, 3, 4, 5, 6, 7, 8 and 9. The data were generated from a single experiment

The genus of polar microalgae was identified via sequence similarity of the small subunit ribosomal DNA sequence (Supplementary rDNA sequence) with the NCBI GenBank database. To determine the individual components and to compare the composition of polar and non-polar microalgae, microalgae extracts were analyzed using GC-MS. The analysis produced 9–13 peaks corresponding to bioactive compounds that were recognized by the retention time and fragmentation pattern through the NIST12.0 spectral library (Fig. 1). Compounds, such as Neophytadiene, 9,12,15-Octadecatrienoic acid (α-linoleic acid), and/or n-Hexadecanoic acid (palmitic acid), that have anti-inflammatory activities [17–19] constituted approximately 60% of the polar and non-polar microalgae extracts, except for the KSF0037 extract that had only 14.72% (Tables 1, 2, 3, 4, 5, 6, 7, 8 and 9). n-Hexadecanoic acid was not detected in non-polar *Micractinium sp*. extracts but represented 18.23% of KSF0015 extract mass. n-Hexadecanoic acid represented 9.531% and methylated Hexadecanoic acid represented 3.421% of KSF0041 extract mass; KSF0015 and KSF0041 are polar microalgae of the same genus (Tables 1, 2 and 3).

**Table 1:** Compounds identified in *Micractinium sp.* extract

	Retention time (min)	Compound	% of total
1	3.825	Cyclotetrasiloxane, octamethyl-	0.83
2	5.620	Cyclotetrasiloxane, decamethyl-	0.343
3	9.335	Pentadecane	1.104
4	10.958	Cyclotetrasiloxane, hexadecamethyl-	0.764
5	12.422	Neophytadiene	14.64
6	13.257	Docosahexaenoic acid methyl ester	6
7	13.329	7,10,13-Hexadecatrienoic acid, (Z,Z,Z)-	8.732
8	13.516	Linoleic acid methyl ester	14.507
9	14.578	Phytol	1.589
10	14.876	9,12,15-Octadecatrienoic acid, (Z,Z,Z)-	46.317
11	21.269	Ergosterol	1.928
12	23.659	Phytyl linolenate	0.952

**Table 2:** Compounds identified in KSF001*5* extract

	Retention time (min)	Compound	% of total
1	9.425	Cycloheptasiloxane, tetradecamethyl-	1.604
2	11.031	Cyclooctasiloxane, hexadecamethyl-	2.679
3	11.080	8-Heptadecene	0.685
4	12.477	Neophytadiene	11.359
5	13.301	Docosahexaenoic acid methyl ester	7.442
6	13.367	7,10,13-Hexadecatrienoic acid, (Z,Z,Z)-	10.808
7	13.558	n-Hexadecanoic acid	18.230
8	14.500	Linolenic acid methyl ester	1.982
9	14.888	9,12,15-Octadecatrienoic acid, (Z,Z,Z)-	34.036
10	15.437	2-Butenedioic acid (E)-, bis(2-ethylhexyl) ester	9.036

**Table 3:** Compounds identified in KSF0041 extract

	Retention time (min)	Compound	% of total
1	11.042	6-Tridecen-4-yne, (Z)-	3.343
2	12.476	Neophytadiene	27.444
3	13.152	Hexadecanoic acid, methyl ester	3.421
4	13.536	n-Hexadecanoic acid	9.531
5	14.497	Linolenic acid methyl ester	8.174
6	14.864	9,12,15-Octadecatrienoic acid, (Z,Z,Z)-	17.484
7	15.432	2-Butenedioic acid (E)-, bis(2-ethylhexyl) ester	9.948
8	17.078	Butyl 9,12,15-octadecatrienoate	3.053
9	18.897	13-Docosenamide, (Z)-	4.014

**Table 4:** Compounds identified in *Chlamydomonas sp.* extract

	Retention time (min)	Compound	% of total
1	3.816	Cyclotetrasiloxane, octamethyl-	0.761
2	11.028	8-Heptadecene	1.467
3	12.431	Neophytadiene	16.65
4	12.777	(Z)-1,3-Phytadiene	4.294
5	13.265	Methyl 4,7,10,13-hexadecatetraenoate	7.861
6	13.519	n-Hexadecanoic acid	15.313
7	14.573	Phytol	1.18
8	14.709	Cyclohexane, 1,2,4-triethenyl-	2.143
9	14.865	9,12,15-Octadecatrienoic acid, (Z,Z,Z)-	27.24
10	17.239	Hexadecanoic acid, 2-hydroxy-1-(hydroxymethyl)ethyl ester	0.594
11	18.378	Linoleic acid, 2-hydroxy-1-(hydroxymethyl)ethyl ester (Z,Z,Z)-	1.855
12	20.805	Vitamin E	0.527
13	21.275	Ergosterol	3.312

**Table 5:** Compounds identified in KNM0029C extract

	Retention time (min)	Compound	% of total
1	9.356	3,4-Difluorobenzoic acid, 4-dodecyl ester	0.736
2	9.420	Cycloheptasiloxane, tetradecamethyl-	0.304
3	10.543	Diphenylamine	0.508
4	12.476	Neophytadiene	19.42
5	13.309	4,7,10,13,16,19-Docosahexaenoic acid, methyl ester, (all-Z)-	13.754
6	13.545	n-Hexadecanoic acid	9.473
7	14.496	Linolenic Acid Methyl Ester	1.492
8	14.776	Omega.-3 Arachidonic Acid methyl ester	7.679
9	14.887	9,12,15-Octadecatrienoic acid, (Z,Z,Z)-	33.280
10	15.430	2-Butenedioic acid (E)-, bis(2-ethylhexyl) ester	5.008
11	18.897	13-Docosenamide, (Z)-	3.510

**Table 6:** Compounds identified in KSF0037 extract

	Retention time (min)	Compound	% of total
1	11.079	8-Heptadecene	1.098
2	12.474	Neophytadiene	5.500
3	12.762	11,11-Dimethyl-spiro[2, 9]dodeca-3,7-dien	3.898
4	12.812	Methyl 4,7,10,13-hexadecatetraenoate	18.807
5	12.882	7,10-Hexadecadienoic acid, methyl ester	6.727
6	13.534	n-Hexadecanoic acid	1.905
7	14.316	Methyl 5,9,12-octadecatrienoate	2.959
8	14.504	Linoleic acid methyl ester	24.965
9	14.871	9,12,15-Octadecatrienoic acid, (Z,Z,Z)-	7.330
10	15.428	2-Butenedioic acid (E)-, bis(2-ethylhexyl) ester	2.002
11	18.898	13-Docosenamide, (Z)-	0.561

**Table 7:** Compounds identified in KSF0134 extract

	Retention time (min)	Compound	% of total
1	11.069	8-Heptadecene	2.379
2	12.476	Neophytadiene	15.537
3	12.826	2-Hexadecen-1-ol, 3,7,11,15-tetramethyl-, [R-[R*,R*-(E)]]-	5.583
4	13.305	(6Z,9Z,12Z,15Z)-Methyl octadeca-6,9,12,15-tetraenoate	13.641
5	13.549	n-Hexadecanoic acid	14.784
6	14.496	Linolenic acid methyl ester	2.945
7	14.882	9,12,15-Octadecatrienoic acid, (Z,Z,Z)-	31.556
8	15.431	2-Butenedioic acid (E)-, bis(2-ethylhexyl) ester	3.52
9	17.074	9,12,15-Octadecatrienoic acid, (Z,Z,Z)-	0.854
10	18.892	13-Docosenamide, (Z)-	1.063

**Table 8:** Compounds identified in *Chlorococcum sp.* extract

	Retention time (min)	Compound	% of total
1	3.818	Cyclotetrasiloxane, octamethyl-	0.761
2	11.024	8-Heptadecene	1.467
3	12.425	Neophytadiene	18.61
4	13.266	Methyl 4,7,10,13-hexadecatetraenoate	7.861
5	13.516	n-Hexadecanoic acid	15.313
6	14.443	Linoleic acid methyl ester	1.18
7	14.572	Phytol	2.143
8	14.872	9,12,15-Octadecatrienoic acid, (Z,Z,Z)-	27.24
9	14.983	Octadecanoic acid	0.513
10	18.374	2-Hydroxy-1-(hydroxymethyl)ethyl (9E, 12E, 15E)-9,12,15-octadecatrienoate	1.855
11	20.801	Vitamin E	0.527
12	21.272	Ergosterol	3.312
13	21.745	Stigmasta-5,7,22-trien-3-ol, (3.beta.)-	2.355

**Table 9:** Compounds identified in KSF0003 extract

	Retention time (min)	Compound	% of total
1	9.422	Cycloheptasiloxane, tetradecamethyl-	1.138
2	11.084	8-Heptadecene	11.195
3	12.252	Tetradecanal	1.244
4	12.479	Neophytadiene	33.039
5	13.155	Hexadecanoic acid, methyl ester	2.924
6	13.544	n-Hexadecanoic acid	11.601
7	14.451	Linoleic acid methyl ester	4.552
8	14.866	9,12,15-Octadecatrienoic acid, (Z,Z,Z)-	16.757
9	15.430	2-Butenedioic acid (E)-, bis(2-ethylhexyl) ester	4.927

Several lipid components, such as Docosahexaenoic acid methyl ester, linolenic acid methyl ester, 13-Docosenamide (Z)-, and methyl 4,7,10,13-Hexadecatetraenoate, were dominant in polar and non-polar extracts (Tables 1, 2, 3, 4, 5, 6, 7, 8 and 9). However, some fatty acids were only found in polar microalgae, such as 4,7,10,13,16,19-Docosahexaenoic acid, methyl ester, (all-Z)- (13.754%) and Omega.-3 arachidonic acid methyl ester (7.679%) in KNM0029C and (6Z,9Z,12Z,15Z)-methyl Octadeca-6,9,12,15-tetradentate in KSF0037 (4.734%) and KSF0134 (13.641%), which were not present in non-polar *Chlamydomonas sp*. (Fig. 1; Tables 4, 5, 6 and 7). In addition, 2-Butenedioic acid I-, bis (2-ethylhexyl) ester, was present at a 3–10% level in all polar microalgae but was not detected in non-polar microalgae extracts. These findings indicate that although the major components of polar and non-polar microalgae are comparable, polar microalgae have distinct profiles of bioactive compounds compared to microalgae from non-polar regions.

### Radical-scavenging activity of microalgae extracts

**Fig. 2:**
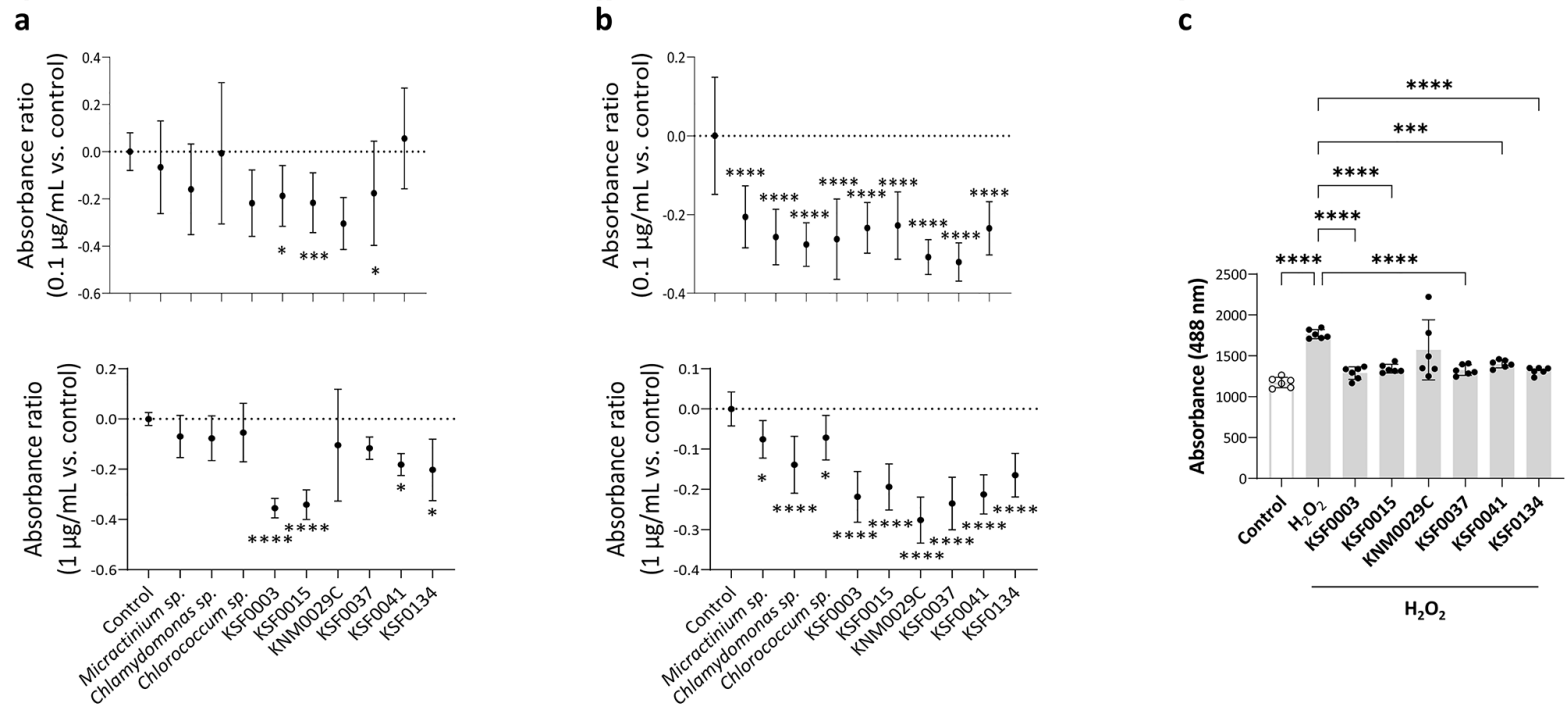
The radical-scavenging activity of the non-polar and polar microalgae extracts. (**a**) Hydrogen peroxide (H_2_O_2_) scavenging activity. The microalgae extracts were incubated with 2′,7′-dichlorofluorescein diacetate (DCHF-DA) and H_2_O_2_ for 10 min. (**b**) Hydroxyl radical-scavenging activity. The microalgae extracts were incubated with DCHF-DA, H_2_O_2_, and FeCl_2_ for 10 min.; n = 12/group. Absorbance ratios were calculated by comparing the fluorescence of the microalgae extract-treated group to that of the control group, which was not treated with microalgae extracts. (**c**) Oxidation of HaCaT cells that were treated with H_2_O_2_ for 18 h after overnight pretreatment with the polar microalgae extracts. In the control group, the HaCaT cells were cultured for 18 h in the absence of H_2_O_2_ and polar microalgae extracts. Data are representative of two independent experiments and presented as the mean ± SD. p-values are determined by one-way ANOVA with Tukey’s multiple comparisons. *p < 0.05, *** p < 0.001, and **** p < 0.0001 vs. control

Microalgae are considered a new potential source of natural antioxidants because synthetic antioxidants can be toxic and most commercially available natural antioxidants come from terrestrial plants [20–22]. To compare the radical-scavenging activities between polar and non-polar microalgae, we measured their abilities to inhibit DCHF-DA fluorescence induced by H_2_O_2_ or hydroxyl radicals (OH.). At a concentration of 0.1 µg/mL, the addition of non-polar and polar algae extracts did not significantly decrease H_2_O_2_- or OH. -induced fluorescence emission, except for KSF0003 and KSF0015 extracts (Fig. 2a). All non-polar and polar algae extracts significantly decreased OH. production at 0.1 µg/mL (Fig. 2b). At a concentration of 1 µg/mL, the addition of KSF0003, KSF0015, and KSF0134 extracts significantly decreased fluorescence emitted by oxidized DCFH (Fig. 2a). A significant decrease in OH. was also observed at 1 µg/mL of non-polar and polar microalgae extracts, but the reduction was more significant in polar microalgae extracts than in non-polar microalgae of the same genus (*Micractinium sp*. vs. KSF0015 p < 0.0001; *Micractinium sp*. vs. KSF0041 p < 0.0001; *Chlamydomonas sp*. vs. KNM0029C p < 0.0001; *Chlamydomonas sp*. vs. KSF0037 p < 0.0006; and *Chlorococcum sp*. vs. KSF0003 p < 0.0001).

To determine if 1 µg/mL of polar microalgae extracts can protect skin epithelial cells against oxidative stress, we assessed their effects on reactive oxygen radical generation in HaCaT cells treated with H_2_O_2_. As shown in Fig. 2c, all polar microalgae extracts significantly decreased reactive oxygen radical generation. These results suggest that polar microalgae extracts have greater radical-scavenging activity than non-polar microalgae extracts. Therefore, polar microalgae extracts may protect skin epithelial cells from oxidative stress-induced damage.

### Polar microalgae extracts improved HaCaT cell viability during stress conditions

**Fig. 3:**
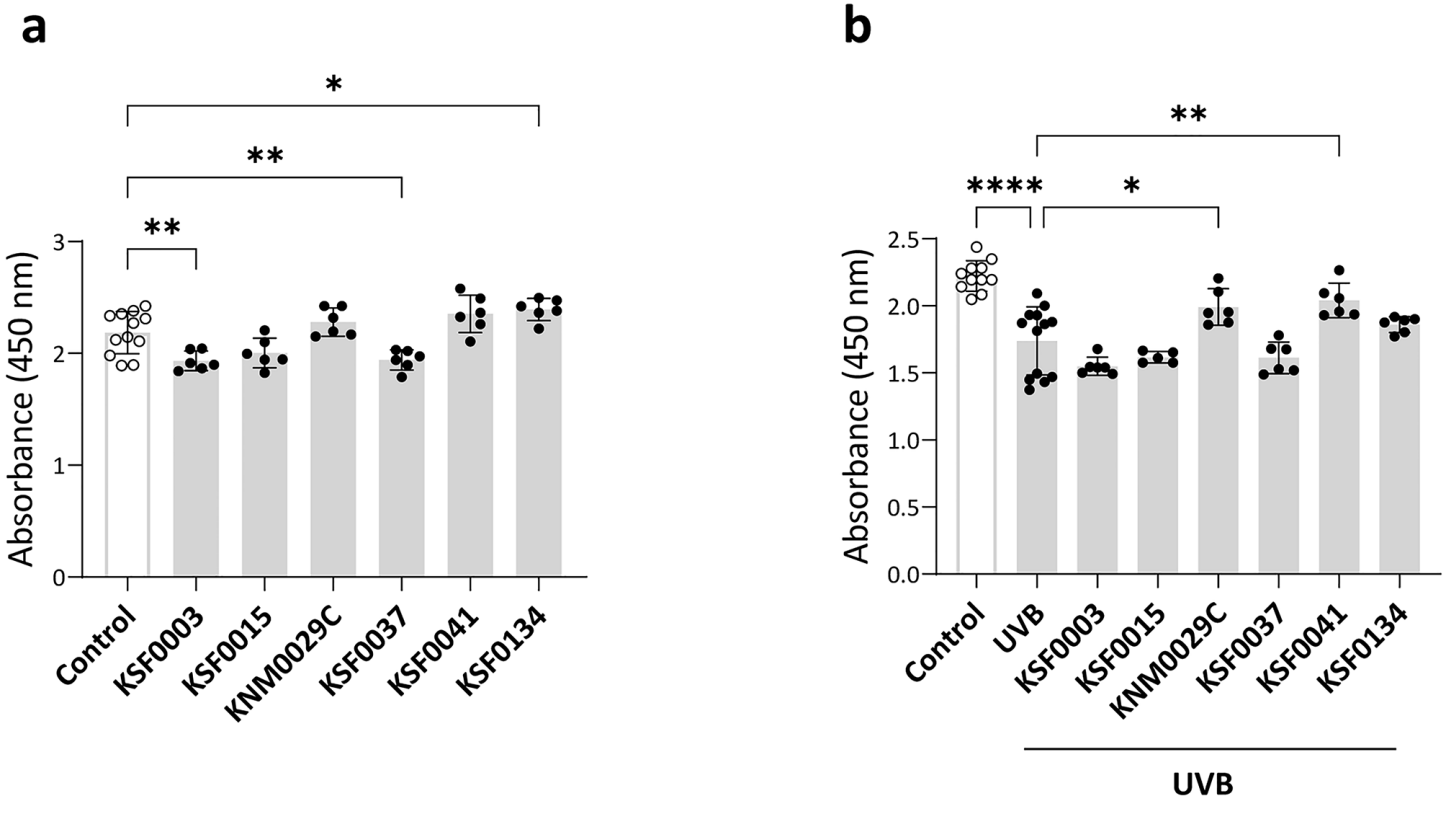
The effects of polar microalgae extract on the viability and intracellular reactive oxygen species scavenging. (**a**) Viability of HaCaT cells treated with or without (control) the polar microalgae extracts for 18 h. (**b**) Viability of HaCaT cells that were pretreated overnight with the polar microalgae extracts, irradiated with ultraviolet (UV) B, and incubated for 18 h. In the control group, the HaCaT cells were cultured for 18 h in the absence of UVB and polar microalgae extracts. Data are representative of two independent experiments and presented as the mean ± SD. p-values are determined by one-way ANOVA with Tukey’s multiple comparisons. * p < 0.05, ** p < 0.01, and **** p < 0.0001

To assess the impact of polar microalgae extracts on HaCaT cell viability, cells were incubated overnight with 1 µg/mL extracts. The addition of KSF0003 or KSF0037 extracts significantly reduced HaCaT cell viability as indicated by a decrease in fluorescence emissions at 450 nm, which was linked to reduced cleavage of tetrazolium salts due to decreased mitochondrial dehydrogenase activity (Fig. 3a). Conversely, the viability of HaCaT cells was unchanged when treated with KSF0015, KNM0029C, or KSF0041 extracts and significantly improved when treated with KSF0134 extracts (Fig. 3a).

To determine the protective effects of polar microalgae extracts on HaCaT cells during damaging stimuli, we measured the viability of UVB-treated HaCaT cells in the presence or absence of the extracts. As shown in Fig. 3b, exposure to 25 mJ/cm^2^ UVB significantly decreased the viability of HaCaT cells. However, when HaCaT cells were pretreated with 1 µg/mL of KNM0029C or KSF0041 extract, viability was significantly increased compared to UVB-treated cells without extract; this viability approached that of UVB-untreated control HaCaT cells (p = 0.0774 for KNM0029C vs. control; p = 0.2878 for KSF0041 vs. control). These findings indicate that polar marine microalgae extracts can protect skin epithelial cells against oxidative stress and radiation-induced damage without causing cytotoxicity in vitro.

### Polar microalgae extracts protected against psoriatic inflammatory stimuli in HaCaT cells

**Fig. 4:**
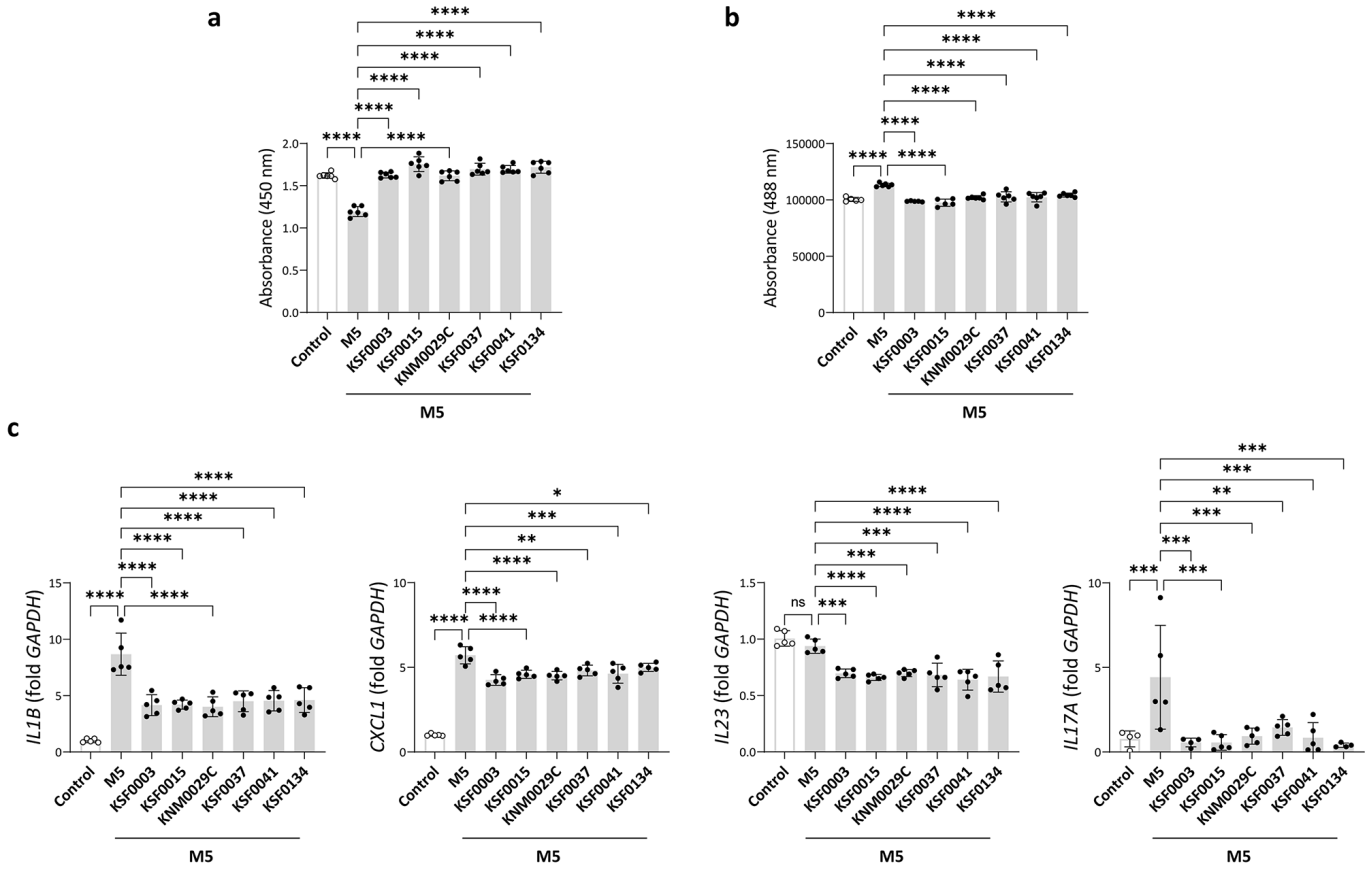
The effects of polar microalgae extracts on the viability, permeability, and expression of inflammatory markers in HaCaT cells treated with M5 cytokines. (**a**) Viability of HaCaT cells that were pretreated overnight with the polar microalgae extracts followed by treatment with M5 cytokines for 18 h. (**b**) Fluorescence of 4-kDa fluorescein isothiocyanate-dextran fluxed from the apical to the basal compartment of transwell chambers seeded with HaCaT cells treated with M5 cytokines for 18 h after overnight incubation with the polar microalgae extracts. (**c**) mRNA expression of *IL1B*, *IL17A*, *IL23*, and *CXCL1* in HaCaT cells treated with M5 cytokines after overnight incubation with the polar microalgae extracts. In the control group, the HaCaT cells were cultured without overnight pretreatment with polar microalgae extracts followed by 18 h of M5 cytokine stimulation. Data are representative of two independent experiments and presented as the mean ± SD. p-values are determined by one-way ANOVA with Tukey’s multiple comparisons. * p < 0.05, ** p < 0.01, *** p < 0.001, and **** p < 0.0001

Psoriasis is a common chronic inflammatory skin disease characterized by abnormal differentiation of keratinocytes and inflammatory changes within the skin layer [23]. Although the pathogenesis of psoriasis is still largely unknown, it is generally accepted that inflammatory mediators released by abnormally functioning keratinocytes stimulate immune cells [23, 24]. To assess the protective activity of polar microalgae extracts on psoriatic keratinocytes, we stimulated HaCaT cells with IL-17 A, IL-22, oncostatin M, IL-1α, and TNF-α (M5 cytokines) [25, 26] in the presence or absence of polar microalgae extracts. Treatment of HaCaT cells for 3 h with M5 cytokines significantly reduced viability, which was reversed by pretreatment with all polar microalgae extracts (Fig. 4a). M5 cytokine stimulation resulted in decreased viability and HaCaT cells showed an increased flux of FITC-labeled dextran indicating impaired barrier integrity (Fig. 4b). However, pretreating HaCaT cells with polar microalgae extracts blocked the increased permeability caused by M5 cytokine treatment (Fig. 4b). In addition, pretreatment of HaCaT cells with polar microalgae extracts significantly decreased expression of *IL1B*, *IL23*, *CXCL1*, and *IL17A* mRNA, which are mediators implicated in the pathogenesis of psoriasis [27], compared to HaCaT cells treated with only M5 cytokines (Fig. 4c).

### Transcriptome changes induced by polar microalgae extracts in HaCaT cells stimulated with psoriatic inflammatory stimuli

**Fig. 5:**
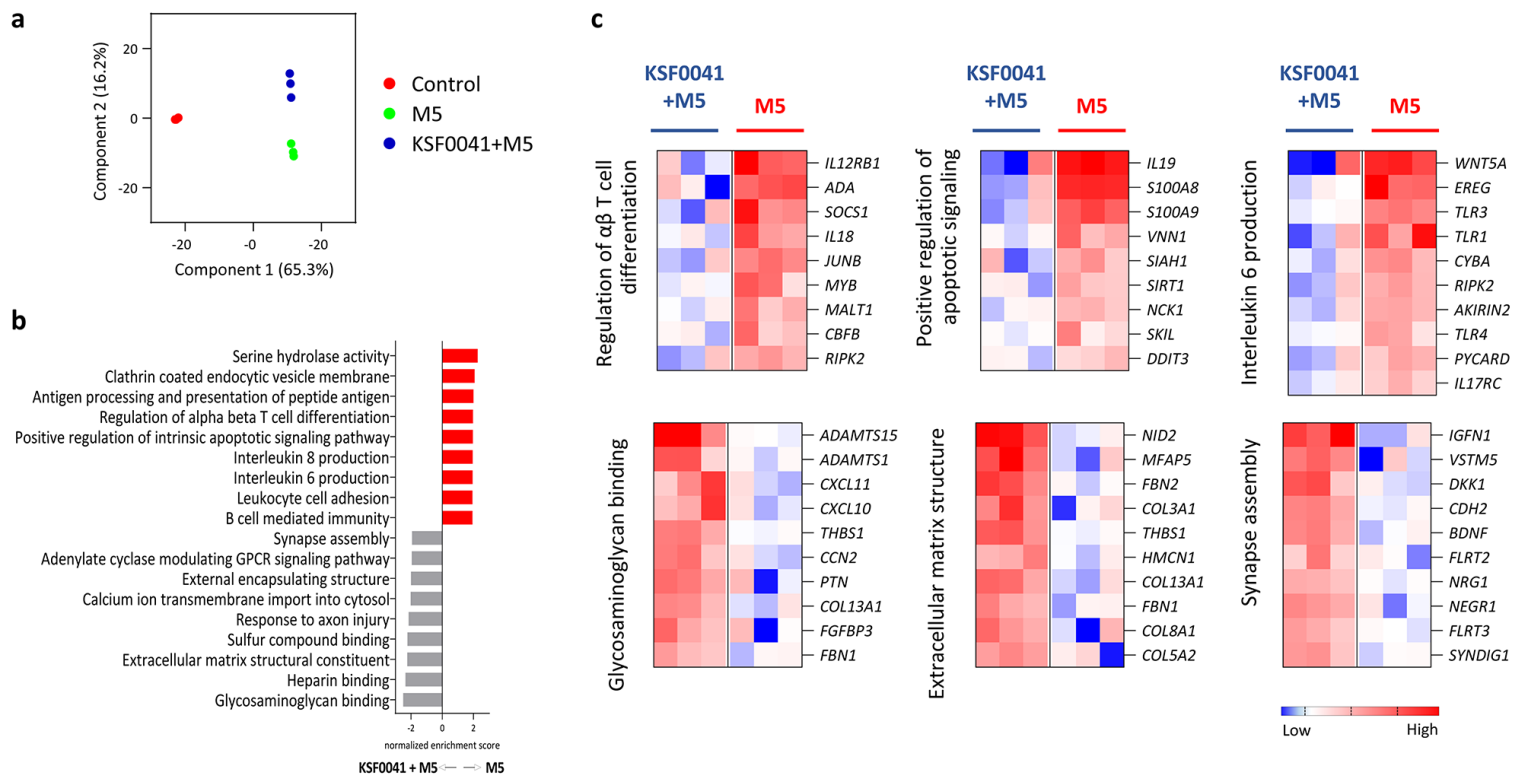
The effects of polar microalgae extracts on the transcriptome of HaCaT cells treated with M5 cytokines. (**a**) Multidimensional scaling analysis of gene expression data. In the control group, the HaCaT cells were cultured without overnight pretreatment with polar microalgae extracts followed by 18 h of M5 cytokine stimulation. (**b**) Gene ontology enrichment in M5 cytokine-stimulated HaCaT cells that were pretreated with or without KSF0041 by gene set enrichment analysis. (**c**) Selected gene sets that were differentially expressed between HaCaT cells pretreated with KSF0041 and stimulated with M5 cytokines and HaCaT cells only stimulated with M5 cytokines (adjusted p < 0.05). Heat map showing the log2 fold changes to the geometric mean of fragments per kilobase of exons per million fragments mapped + 0.01. The data were generated from a single experiment

KSF0041 extract was the only polar microalgae extract that showed radical-scavenging activity and protected HaCaT cells against UVB and M5 cytokine-induced damage without reducing cell viability (Figs. 2, 3 and 4). To gain a deeper understanding of the cytoprotective effects of KSF0041 extract, we compared the transcriptome of untreated M5 cytokine-stimulated HaCaT cells to those treated with KSF0041 extract. The multidimensional scaling indicated that the control, KSF0041 extract-treated M5 cytokine-stimulated, and M5 cytokine-stimulated HaCaT cells segregated into three different groups (Fig. 5a), implying marked transcriptomic changes in KSF0041 extract-treated M5 cytokine-stimulated HaCaT cells.

GSEA identified differentially enriched Gene Ontology (GO) terms in M5 cytokine-stimulated HaCaT cells treated with or without KSF0041 extract (Fig. 5b). Most enriched GO terms in the M5 cytokine-stimulated HaCaT cells were related to inflammatory responses, such as regulation of αβ T-cell differentiation, positive regulation of apoptotic signaling, and IL-6 production (Fig. 5b and c). Additionally, the upregulated expression of acute inflammatory mediators (*S1008A* and *S100A9*), inflammasome adaptor protein (*PYCARD*), and IL-17 class cytokine (*IL17C*) in M5 cytokine-stimulated HaCaT cells indicated activated psoriatic inflammatory responses. However, in M5 cytokine-stimulated HaCaT cells treated with KSF0041 extract, the enriched gene sets shifted to those related to physiologic functions, such as glycosaminoglycan binding, extracellular matrix structure, and synapse assembly (Fig. 5b and c). These results indicate that KSF0041 extract treatment attenuated inflammatory responses in M5 cytokine-stimulated HaCaT cells while recovering normal cellular functions.

### KSF0041 extract ameliorated psoriatic skin inflammation in mice

**Fig. 6:**
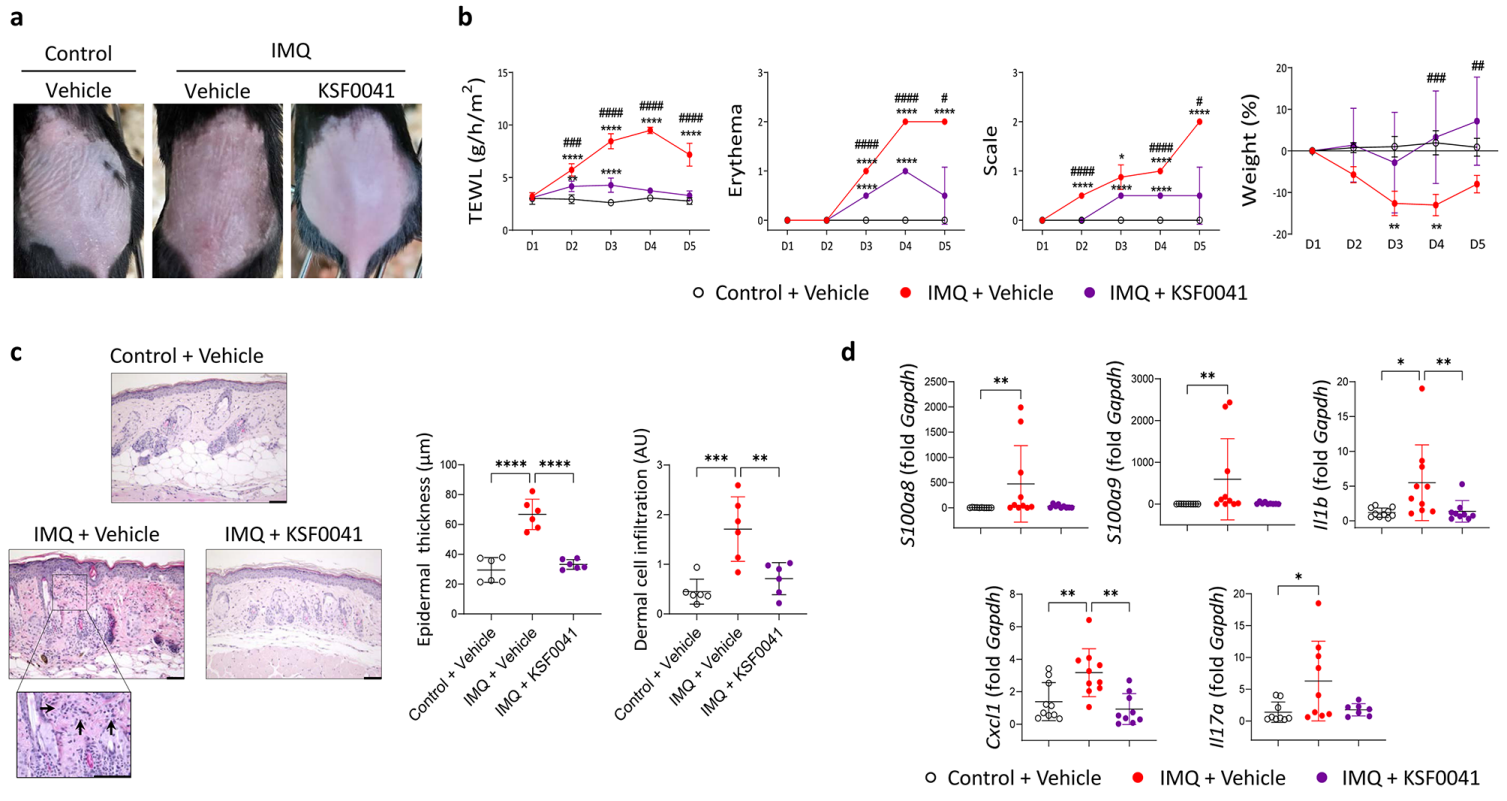
The effects of topical application of KSF0041 extract on psoriatic skin inflammation in mice induced by imiquimod. (**a**) Macroscopic findings for back skin on day 5 in control and imiquimod (IMQ)-applied mice with or without KSF0041 treatment. (**b**) Transepidermal water loss (TEWL), erythema, scale, and weight change of mice (n = 4–5/experimental group). Data are representative of two independent experiments. (**c**) Hematoxylin and eosin-stained images (left), epidermal thickness (middle), and dermal cell infiltration (right) of skin. The quantification data were generated by pooling data from two independent experiments. Scale bars, 62.3 μm. Arrows indicate representative cell infiltration. (**d**) *S100a8*, *S100a9*, *Il1b*, *Cxcl1*, *and Il17a* mRNA expression in the skin. *Gapdh* served as the internal control. Data are representative of two independent experiments. In the control group, mice were treated with Vaseline and vehicle of KSF0041 cream. Data are presented as the mean ± SD. p-values are determined by two-way ANOVA with Bonferroni’s multiple comparisons (b) or one-way ANOVA with Tukey’s multiple comparisons (c and d). * p < 0.05, ** p < 0.01, and **** p < 0.0001 vs. control. # p < 0.05, ## p < 0.01, ### p < 0.001, and #### p < 0.0001 IMQ + Vehicle vs. IMQ + KSF0041.

To evaluate the efficacy of KSF0041 extract in treating psoriasis in vivo, we treated mice with psoriatic dermatitis induced by dermal application of imiquimod [28] with topical KSF0041 extract. Imiquimod treatment induced clinical features of psoriasis, such as erythema and scales, and increased transepidermal water loss (TEWL) and weight loss (Fig. 6a and b). However, the topical application of KSF0041 extract to imiquimod-treated mice improved the macroscopic features of psoriatic inflammation, alleviated weight loss, and reduced the weights of the spleen and draining axillary lymph nodes, which indicate attenuated psoriatic inflammatory responses (Fig. 6a and b; Fig. S1). Histopathological analysis showed a marked decrease in dermal cell infiltration, epidermal thickness, and hyperkeratosis in the KSF0041 extract- and imiquimod-treated group compared to the imiquimod-only group; thus, the KSF0041 extract attenuated the dermal infiltration of inflammatory immune cells (Fig. 6c). Accordingly, the topical treatment of KSF0041 extract decreased mRNA expression of innate immune mediators (*S100a8*, *Il1b*, and *Il23*) and *Il17a* involved in psoriasis in the lesional skin of psoriatic dermatitis-induced mice (Fig. 6d).

## Discussion

Six polar microalgae, belonging to *Micractinium sp*., *Chlamydomonas sp*., or *Chlorococcum sp*., contain various bioactive compounds that are distinct from those of their non-polar counterparts within the same genus. We demonstrated that extracts from these polar microalgae can protect skin from oxidative stress, UVB radiation, and psoriatic skin inflammation. Microalgae offer many advantages for developing bioactive molecules due to their biodiversity, productivity, and metabolic plasticity under various culture conditions [10, 29]. Microalgae in polar regions have adapted to the extreme and oscillating polar environment that includes freezing temperatures and oxidative and nutrient stresses [7, 30]. However, the composition, biological activities, and therapeutic potential of polar microalgae have not been fully investigated. Although our analysis showed that polar and non-polar microalgae extracts share major compounds that have anti-inflammatory activities, polar microalgae have distinct chemical profiles compared to non-polar microalgae of the same genus. The differences in compound profiles were prominent in the fatty acid composition as shown by the presence of 13-Docosenamide (Z)-, which has antinociceptive and anti-inflammatory activities [31], in KSF0041, KNM0029C, KSF0037, and KSF0134 extracts, but not in extracts of their non-polar *Micractinium sp*. and *Chlamydomonas sp*. counterparts. Furthermore, 4,7,10,13,16,19-Docosahexaenoic acid (DHA) methyl ester, (all-Z)-, an analog of Omega.-3 fatty acid DHA [32], was present in KNM0029C extract and (6Z,9Z,12Z,15Z)-methyl Octadeca-6,9,12,15-tetraenoate was present in KSF0037 and KSF0134 extracts; these were not found in extracts of their non-polar *Chlamydomonas sp*. counterparts. Polar microalgae must maintain cell membrane fluidity to transport nutrients and metabolic waste at low temperatures [7]. Thus, it is likely that they have altered their fatty acid composition to adapt to freezing temperatures. KSF0041 contains a more complex fatty acid composition than the non-polar *Micractinium sp*., which has a relatively simple composition of alpha-linoleic acid or its derivatives. The composition of KSF0041 includes n-Hexadecanoic acid, Hexadecanoic acid, methyl ester, Methyl 4,7,10,13-hexadecatetraenoate, Butyl 9,12,15-octadecatrienoate, and 13-Docosenamide, (Z)- in addition to alpha-linoleic acid and linoleic acid methyl ester.

The solubility of oxygen increases at low temperatures resulting in hyperoxic environments that are susceptible to the formation of reactive oxygen species (ROS) [7]. To survive abundant ROS, polar microalgae have robust antioxidant systems, as evidenced by elevated levels of superoxide dismutase [33, 34]. Skin, the largest organ in our body, is susceptible to oxidative stress due to continuous exposure to air pollutants, xenobiotics, and UV radiation, all of which promote ROS generation [35]. Therefore, we examined whether pretreatment with polar microalgae extracts could protect HaCaT cells from oxidative stress. With enhanced radical-scavenging activity compared to extracts from their non-polar microalgae counterparts, polar microalgae extracts reduced oxidative stress generated in HaCaT cells. KNM0029C and KSF0041 extracts prevented the reduced viability of HaCaT cells caused by UVB radiation, suggesting that these polar microalgae contain cytoprotective substances for skin epithelial cells.

Psoriasis is a chronic inflammatory skin disease caused by genetic and environmental factors, resulting in epidermal hyperplasia and dermal infiltration of immune cells [36]. While previously considered an immune cell-mediated disease with a key pathogenic role of IL-23/IL-17 [37], recent evidence indicates that keratinocytes also play a role in triggering psoriasis by releasing chemokines and antimicrobial peptides that induce innate inflammation [38]. Considering side effects and efficacy loss of biologics targeting IL-23 or IL-17 [38], therapeutics that can attenuate inflammatory responses in keratinocytes could be promising for psoriasis treatment. Our study found that polar microalgae extracts counteract psoriatic changes in keratinocytes induced by an M5 cytokine cocktail that causes cutaneous inflammation [25]. Polar microalgae extracts prevented reduced viability and barrier integrity in M5 cytokine-stimulated HaCaT cells. M5 cytokine cocktail stimulation for 24–72 h induced proliferation of HaCaT cells (Fig. S2a), as previously described [26]. Therefore, the reduced viability of M5 cytokine-stimulated HaCaT cells observed in this study may be attributed to the 3-h culture period, which was selected on the basis of the increase in inflammatory cytokines in stimulated HaCaT cells (Fig. S2b). The biosynthesis of epidermal ceramides, cholesterol, and fatty acids promotes repair of the disrupted barrier [39]. Therefore, we suggest that the presence of abundant and diverse fatty acids in polar microalgae extracts may play a role in epithelial protection, considering fatty acids promote epithelial preservation and barrier integrity [40, 41]. Polar microalgae extracts also prevented the increased expression of inflammatory cytokines involved in psoriasis. GSEA of differentially regulated genes in M5 cytokine-stimulated HaCaT cells showed that treatment with KSF0041 extract, selected because of its protective effect against oxidative stress, UVB radiation, and M5 cytokine stimulation, shifted the transcriptome from an inflammatory response toward normal physiologic functions. KEGG pathway analysis of the genes upregulated in M5 cytokine-stimulated HaCaT cells showed enrichment of key pathways implicated in psoriatic inflammation, such as cytokine-cytokine receptor interaction, IL-17 signaling pathway, and TNF signaling pathway (Fig. S3). Although KSF0041 extract was unable to downregulate all upregulated genes in M5 cytokine-stimulated HaCaT cells, it downregulated expression of epidermal pathogenesis mediators (*S100A7*, *S100A8*, and *S100A9*) [42] and a potential biomarker for cells undergoing programmed cell death (*PTGS2*) [43]. We further evaluated the therapeutic potential of KSF0041 extract on an imiquimod-induced psoriasis-like mouse model. Topical application of KSF0041 extract almost completely alleviated the clinical features of psoriasis, such as scaling, redness, and loss of epithelial water and weight. The treatment also prevented pathogenic histological findings and upregulated lesion expression of inflammatory cytokines. Collectively, these results suggest that polar microalgae are promising natural resources for protecting skin from harmful damage and pathogenic inflammation. Several mouse models of psoriasis are available, each with varying degrees of recapitulating the clinical features of psoriasis [44]. Notably, K14-ARGE transgenic mice, which express human amphiregulin in the basal epidermis under human keratin 14-promoter [45], and K5.Stat3C mice, which harbor a constitutively activated form of Stat3 under the control of bovine K5 regulatory sequence [46], exhibit psoriasis-like skin phenotypes due to abnormal activation of keratinocytes. While imiquimod can induce epidermal expression of IL-17/IL-23 inflammation mediators [47], assessing the therapeutic effects of polar microalgae on these mouse models, characterized by aberrant keratinocyte activity, would allow us to gain insight into the effect of polar microalgae in protecting keratinocytes.

The identification of individual compounds with specific bioactivities is required for the pharmaceutical application of natural resources. As part of this approach, we isolated three individual alkaloid fractions of KSF0041 extract using a thin-layer chromatogram and evaluated the composition and bioactivity of each fraction (Fig. S4). The chromatogram of each KSF0041 extract fraction showed a unique composition compared to that of the total extract (Fig. S4a and Table S2-S4). However, the bioactivity of the KSF0041 alkaloid fraction was comparable to that of the KSF0041 total extract in terms of restoring decreased viability and inhibiting FITC-dextran flux and the expression of inflammatory cytokines in M5 cytokine-stimulated HaCaT cells (Fig. S4c–e). These results suggest that the inhibition of psoriatic inflammation, at least partially, comes from the synergistic effect of compounds in the KSF0041 extract rather than an individual compound.

Considering that the polar microalgae we tested can be cultured at low temperatures, these polar microalgae are promising as a source of substances with radical-scavenging and anti-inflammatory properties that can provide skin barrier protection. Further research is needed to isolate and identify the bioactive compounds from polar microalgae, which will enable their potential use as therapeutic agents against inflammatory skin diseases.

## Conclusions

Our study demonstrated that six polar microalgae species, belonging to the genera *Micractinium sp*., *Chlamydomonas sp*., or *Chlorococcum sp*., have unique bioactive compounds with skin protective properties against oxidative stress, UVB radiation, and psoriatic skin inflammation. The differences in compound profiles between polar and non-polar microalgae were notable, especially in fatty acid composition; this may be due to the adaptation of polar microalgae to the extreme polar environment. Polar microalgae extracts also have better radical-scavenging activity than non-polar microalgae extracts, and they were able to protect human keratinocyte cells against oxidative stress and UVB radiation. Polar microalgae extracts also inhibited the increase in inflammatory cytokines involved in psoriasis. Furthermore, KSF0041 extract, selected based on its ability to protect HaCaT cells against oxidative stress, UVB, and an inflammatory cytokine cocktail showed a therapeutic effect in an imiquimod-induced psoriasis-like mouse model. These findings highlight the potential of polar microalgae as a source of bioactive compounds for skin protection and suggest that they could be promising candidates for the development of novel therapeutic agents for psoriasis.

## Materials and methods

### Sample collection and identification

The polar microalgae samples KSF0003, KSF0015, KSF0037, and KSF0041 were collected in 2006 and KSF0134 was collected in 2014 from freshwater in Byers Peninsula, Livingston Island in Antarctica (S 62 40’09.81” W 61 06’26.61”). KNM0029C was obtained from seawater in the vicinity of Dasan Station in Ny-Ålesund, Spitsbergen, Norway (78°55′N, 11°56′E) in 2008. All microalgae samples were deposited at the Korea Polar Research Institute located in the Republic of Korea and stored at 2–3 °C under a white light source from light-emitting diodes (35 µmol·m^− 2^·s^− 1^). Based on small subunit ribosomal DNA sequence similarities within the NCBI GenBank database, KSF0015 and KSF0041 were identified as belonging to the genus *Micractinium sp*., KNM0029C, KSF0037, and KSF0134 were determined to be of the genus *Chlamydomonas* sp., and KSF0003 was found to be of the genus *Chlorococcum sp*. As control strains, *Micractinium sp*. (LIMS-PS-1788), *Chlamydomonas* sp. (LIMS-PS-0082), and *Chlorococcum* sp. (LIMS-PS-0085), isolated from moderate-temperature regions, were obtained from the Library of Marine Samples (LIMS) of the Korea Institute of Ocean Science and Technology.

### Microalgae culture

KSF0015 and KSF0041 were cultured on Bold’s Basal Medium, and KSF0003, KSF0037, KSF0134, and KNM0029C were cultured on Tris-acetate-phosphate (TAP) medium [48]. All strains were grown at 11–12 °C for 4 weeks under controlled conditions in a 15-L photo-bioreactor with white LED lights at 80 µmol photon m^− 2^s^− 1^ on a 16:8 h light-dark cycle and supplied with filtered air at a rate of 5 L/min [49]. *Micractinium* sp. (LIMS-PS-1788) was cultured in BG-11 medium, and *Chlamydomonas* sp. (LIMS-PS-0082) and *Chlorococcum* sp. (LIMS-PS-0085) were cultured in 1 L of TAP medium with the same light conditions at 24 °C and supplied with filtered air at a rate of 0.5 L/min. Biomass was harvested through centrifugation (15 °C, 30 min, 12,000 rpm) and dried in a freeze-dryer to remove moisture.

### Extract preparation

Microalgae extract was prepared following a previously described method with minor modification [12]. Dried microalgae material (10 g) was suspended in 40 mL of 80% methanol in 50-mL conical tubes. Conical tubes were incubated on a reciprocating shaker for 24 h with continuous agitation at 150 rev/min to dissolve bioactive compounds. The conical tubes were then centrifuged (4 °C, 15 min, 4000 rpm) and supernatants were filtered through Whatman no. 1 filter paper. A rotary vacuum evaporator with a water bath temperature of 37 °C was used to remove the solvents. Residues were collected and used for the experiments.

### Gas chromatography-mass spectrometry (GC-MS) analysis

GC-MS analysis was performed using an Agilent 7890 B gas chromatograph equipped with a 5977B mass selective detector (GC/MSD) (Agilent Technologies, Santa Clara, CA, USA). Chromatographic separation was achieved using a Rxi-5HT capillary column (30 m × 0.25 mm I.D., 0.25 μm film thickness; RESTEK, Bellefonte, PA, USA). An autosampler (Agilent Technologies; 7683B) was used for all experiments. The temperature of the injector was 280 °C. Using split mode, 1 µL of each extract was injected at a ratio of 1/50. The carrier gas was helium C-60 at a constant flow rate of 1 mL/min. The GC oven temperature was initially 60 °C for 1 min, increased to 160 °C in 3 °C/min increments, and then increased to 320 °C in 10 °C/min increments, followed by a hold for 10 min. The mass spectrometer used the electron ionization mode at 70 eV with the ion source temperature set at 250 °C. The running time was 30 min. Scan mode was used in the range of 30–600 m/z with a scan rate of 2.6 scan/s. Agilent Mass Hunter Qualitative Analysis B.04.00 software was used for data analysis. Single compounds were identified by comparing mass spectra with NIST mass spectral libraries (National Institute of Standards, 2020 version) and the Wiley Registry 12th Edition.

### Cell culture

HaCaT cells were cultured at 37 °C in a 5% CO_2_ atmosphere in Dulbecco′s Modified Eagle′s Medium (DMEM)-high glucose, supplemented with 10% (v/v) fetal bovine serum, 1% (v/v) penicillin-streptomycin, 1 M hydroxyethyl piperazineethanesulfonic acid, 100 mM sodium pyruvate, 50 mM 2-mercaptoethanol, 10 mM non-essential amino acids, and L-glutamine. For inflammatory stimuli, HaCaT cells were irradiated with 25 mJ/cm^2^ UVB and then cultured for 4 h to induce epithelial damage. For the psoriatic keratinocyte model, HaCaT cells were stimulated with a final concentration of 2.5 ng/mL recombinant IL-17 A, IL-22, oncostatin M, IL-1α, and TNF- α (Peprotech, Rocky Hill, NJ, USA) for 3 h, as previously described [25, 26].

### Radical-scavenging assay

Microalgae extracts (0.1 µg/mL or 1 µg/mL) and 50 µM 2′,7′-dichlorofluorescein diacetate (DCHF‐DA) (Sigma–Aldrich, St. Louis, MO, USA) were incubated with 10 mM hydrogen peroxide (H_2_O_2_) (Sigma–Aldrich) or 60 mM H_2_O_2_ and 0.75 mM FeCl_2_ in pH 7.4 phosphate-buffered saline (PBS) at 37℃ for 10 min [50]. All experiments were performed in a dark room to prevent oxidation of the DCHF-DA. Fluorescence quenching was measured using a spectrofluorometer (VICTOR3, PerkinElmer, Waltham, MA, USA) with excitation and emission wavelengths of 488 and 530 nm, respectively.

### Cell-based ROS scavenging assay

Cell-based ROS scavenging assay was performed using a previously reported method with minor modification [50]. HaCaT cells were plated in 96-well black plates at a density of 1 × 10^4^ cells/well in 100 µL MEM and incubated for 24 h. The antioxidant activity of the microalgae extracts was assessed by adding 250 mM H_2_O_2_ after the cells were first overnight incubated with the polar microalgae extracts (1 µg/mL). Cells were then washed with pH 7.4 PBS and incubated for 30 min with pH 7.4 PBS containing 10 µM DCHF-DA. Fluorescence was measured using a spectrofluorometer (VICTOR3) with excitation and emission wavelengths of 485 and 535 nm, respectively.

### Cell viability assay

The effect of microalgae extracts on the viability of HaCaT cells was determined using a water-soluble tetrazolium salt assay kit according to the manufacturer’s instructions (EZ Cytox cell viability assay kit; DoGEN Bio, Seoul, Korea). Absorbance was measured at 450 nm (VICTOR3).

### Fluorescein isothiocyanate (FITC)-dextran permeability assay

HaCaT cells were seeded on tissue culture polycarbonate membrane filters (pore size, 0.4 μm) in 24-well transwell plates (SPL Life Sciences, Gyeonggi-do, Korea) at a seeding density of 1.0 × 10^5^ cells/cm^2^. Epithelial permeability across HaCaT monolayers was assessed by measuring the flux of 4-kDa FITC-labeled dextran (2 mg/mL) (Sigma–Aldrich) from the apical chamber to the basolateral chamber of the transwells [9]. FITC-dextran was added to the apical chamber and incubated for 2 h at 37 °C. Fluorescence in the basolateral compartment was then measured at an excitation of 485 nm and emission of 535 nm (VICTOR X4, PerkinElmer).

### Real-time polymerase chain reaction (PCR) analysis

Total RNA was extracted using QIAzol® lysis reagent (Qiagen, Hilden, Germany) and subsequently column purified using an RNeasy® Mini Kit (Qiagen). The RNA (500 ng) was then treated with DNase I (New England Biolabs, Ipswich, MA, USA), and cDNA was synthesized using an iScript™ cDNA synthesis kit (Bio-Rad Laboratories, Hercules, CA, USA). Real-time PCR was performed using iQ SYBR® Green Supermix (Bio-Rad Laboratories) on a CFX Connect™ real-time PCR detection system (Bio-Rad Laboratories). Primers are listed in Table S1.

### RNA extraction, library construction, and sequencing

RNA was extracted from HaCaT cells using QIAzol® and purified using an RNeasy® mini kit (Qiagen). Purified RNA was processed with DNase I (New England Biolabs) to remove genomic DNA. Total RNA concentrations were calculated using Quant-IT RiboGreen (Invitrogen, Waltham, MA, USA). To assess the integrity of the total RNA, samples were run on a TapeStation RNA ScreenTape (Agilent). Only high-quality RNA preparations with RNA integrity number greater than 7.0 were used for RNA library construction. Libraries were independently prepared with 1 µg of total RNA for each sample using an Illumina TruSeq Stranded mRNA Sample Prep Kit (Illumina, San Diego, CA, USA). The libraries were quantified using KAPA Library Quantification kits for Illumina Sequencing platforms according to the qPCR Quantification Protocol Guide (Kapa Biosystems, Wilmington, MA, USA) and qualified using a TapeStation D1000 ScreenTape. Indexed libraries were then submitted to an Illumina NovaSeq, and paired-end (2 × 100 bp) sequencing was performed by Macrogen (Seoul, Korea).

### Sequence annotation and statistical analysis of gene expression

Raw reads were preprocessed from the sequencer to remove low-quality and adapter sequences before analysis; processed reads were aligned with *Homo sapiens (GRCh38)* using HISAT v2.1.0 [51]. The relative gene abundance was measured in Read Count using StringTie [52, 53]. Genes with one more than the zeroed Read Count value in the samples were excluded. To facilitate log2 transformation, 1 was added to each Read Count value of each filtered gene. Filtered data were log2-transformed and subjected to relative log expression normalization. Statistical significance of the differential expression data was determined using the nbinomWaldTest using DESeq2 and fold change in which the null hypothesis was that no difference existed among groups. The false discovery rate (FDR) was controlled by adjusting the p-value using the Benjamini-Hochberg algorithm.

### Multidimensional scaling (MDS)

To visualize the similarities among samples, MDS was used, which converts the structure in a similarity matrix to a simple geometrical picture as scatter plots. The larger the dissimilarity between two samples, the further apart the points representing the experiments in the picture should be. Euclidean distance was applied as the measure of dissimilarity.

### Gene set enrichment analysis (GSEA)

GSEA was performed using GSEA v4.2.3 software provided by the Broad Institute (Cambridge, MA, USA) as previously described [54]. Enrichment analysis was performed using hallmark gene sets of the MsigDB database. To determine the enrichment of ontology gene sets (C5.all.v2022.1), mouse gene symbols were remapped to human orthologs. Selected gene sets with p < 0.05 and FDR < 0.25 are shown.

### Mice

Six-week-old, female, specific pathogen-free C57BL/6 mice were purchased from Orient Bio (Gyeonggi-do, Korea). Female mice were selected because of their robust inflammatory response against psoriatic inflammation induced by TLR7 application [55, 56]. Mice were maintained under standard temperature and humidity in specific pathogen-free conditions. All procedures involving mice were reviewed and approved by the Center of Animal Care and Use of the Lee Gil Ya Cancer and Diabetes Institute, Gachon University (Number: LCDI-2023-0017).

### Animal model of psoriasis-like skin inflammation and KSF0041 extract treatment

Mice received five sequential daily topical doses of 62.5 mg of imiquimod cream (5%) (Aldara™; 3 M Pharmaceuticals, Maplewood, MN, USA) or Vaseline cream (Unilever, Rotterdam, Netherlands) on their shaved back, as described [28]. Mice were assessed for body weight. TEWL was measured on the back skin using a Tewameter TM 210 (Courage + Khazaka GmbH, Cologne, Germany). The composition of KSF0041 cream (with 1% KSF0041 extract) was as follows: 50 mg KSF0041 extract, 0.9 g glycerin, 3 g olive oil, and 1.2-2.4 g olive oil wax. The mixture was incubated overnight at room temperature and stored at 4 °C. A daily topical dose of 62.5 mg KSF0041 cream or vehicle cream (without KSF0041) was applied daily during the imiquimod application period. The mice were euthanized by carbon dioxide asphyxiation 5 days after induction of psoriasis-like skin inflammation.

### Histology

Skin specimens were fixed in 10% neutral buffered formalin (BBC Biochemical, Mount Vernon, WA, USA) and embedded in paraffin. Six 4-µm sections per experimental group were stained with hematoxylin and eosin and visualized using a DM6 B microscope with a DFC7000T camera (Leica, Wetzlar, Germany). Epidermal thickness was quantified by analyzing and averaging images from three distinct regions of each section. Additionally, each section was analyzed to quantify dermal cell infiltration using i-SOLUTION™ (IMT i-Solution Inc., Vancouver, BC, Canada).

### Statistical analysis

All experiments were performed in duplicate. Differences between groups were examined for statistical significance using one-way ANOVA with the Tukey post hoc test. Multiple-comparison tests were performed using two-way ANOVA with Bonferroni’s post hoc test. A p-value < 0.05 was considered statistically significant. GraphPad Prism 9 (GraphPad, San Diego, CA, USA) was used for data analysis. There were no studies in which the investigators were blinded.

## Electronic supplementary material

Below is the link to the electronic supplementary material.

Supplementary Material 1

Supplementary Material 2

## Data Availability

The datasets generated and/or analyzed during the study are available from the corresponding author upon reasonable request.

## List of abbreviations

UV: Ultraviolet
TAP: Tris-acetate-phosphage
GC-MS: Gas chromatography coupled with a mass spectrometer
DCHF-DA: 2′,7′-dichlorofluorescein diacetate
H_2_O_2_: Hydrogen peroxide
PBS: Phosphate-buffered saline
FITC: Fluorescein isothiocyanate
PCR: Polymerase chain reaction
FDR: False discovery rate
MDS: Multidimensional scaling
GSEA: Gene set enrichment analysis
TLC: Thin-layer chromatography
OH: Hydroxyl radical
GO: Gene Ontology
TEWL: Transepidermal water loss
DHA: Docosahexaenoic acid
ROS: Reactive oxygen species
