# Supplementary material for: Polar microalgae extracts protect human HaCaT keratinocytes from damaging stimuli and ameliorate psoriatic skin inflammation in mice
Source: Biol Res. 2023 Jul 13;56:40. doi: 10.1186/s40659-023-00454-1 (PMC10339489; doi:10.1186/s40659-023-00454-1)
Supplement: Supplementary file 2 — Supplementary Material 2 [file 40659_2023_454_MOESM2_ESM.docx]

## Supplementary Information

**Thin-layer chromatography (TLC)**

The presence of alkaloid compounds was qualitatively assessed using TLC, as previously described [1]. Methanolic extracts of each sample were spotted on pre-coated silica gel 60 F264 plates (Merck, Rahway, NJ, USA). The solvent system used for the separation of alkaloids was a mixture of methanol:demineralized water:ethyl acetate (16.5:13.5:100). After the separation of bioactive compounds, Dragen-Dorff reagents (Merck Millipore, Burlington, MA, USA) were used to identify the alkaloids compounds. The desired lanes were scrapped out of the silica gel and incubated at 4℃ overnight in a chloroform:methanol (2:1) solution. Supernatants were blown with nitrogen gas and reconstituted in 5% DMSO solution at 10 mg/mL.

## 2. Supplementary Figure

##
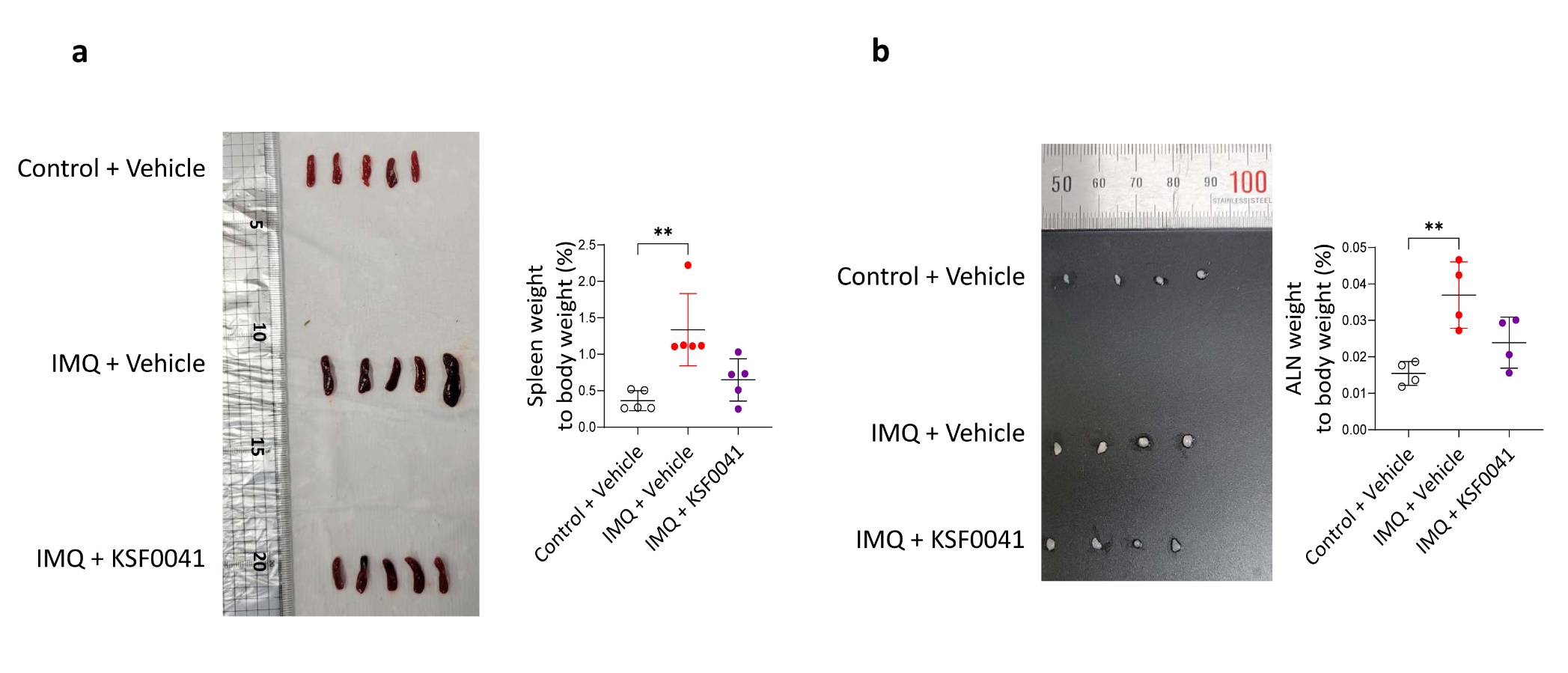


**Figure S1**. **The effects of topical application of KSF0041 extract on the weight of the spleen and draining axillary lymph node in mice with imiquimod-induced psoriatic inflammation.** (a) Representative photograph of the spleen (left) and the percentage of spleen weight to body weight (right). (b) Representative photograph of the axillary lymph node (ALN; left) and the percentage of ALN weight to body weight (right). Data are presented as the mean ± SD. p-values are determined by Kruskal–Wallis test with Dunn’s multiple comparisons (a) or one-way ANOVA with Tukey's multiple comparisons (b). ** p < 0.01.

**
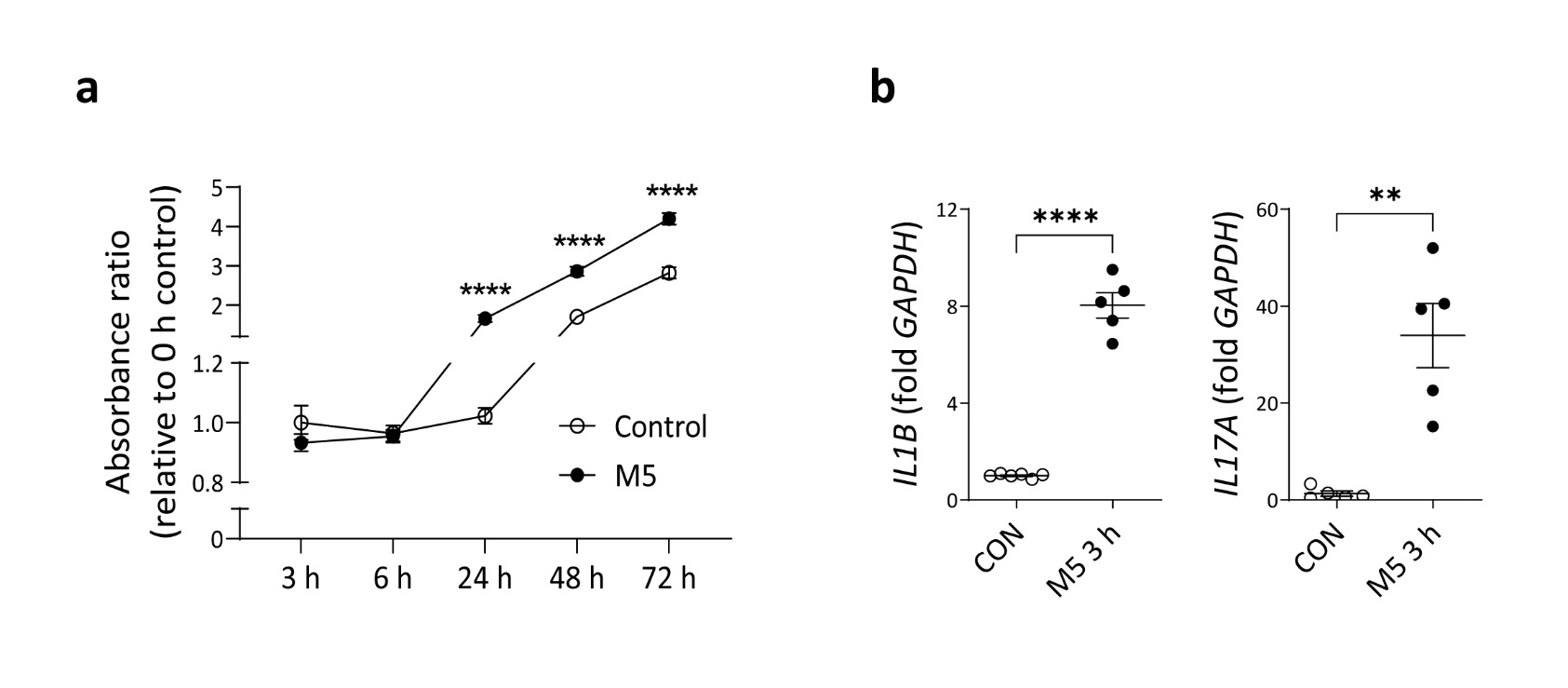
**

**Figure S2**. **The effects of M5 cytokine stimulation on the viability and expression of inflammatory cytokines in HaCaT cells.** (a) Viability of HaCaT cells that were treated with or without M5 cytokines. (b) mRNA expression of *IL1B* and *IL17A* in HaCaT cells treated with M5 cytokines for 3 h. Data are presented as the mean ± SD. p-values are determined by two-way ANOVA with Bonferroni’s multiple comparisons (a) or two-tailed unpaired Student's *t*-test (b). ** p < 0.01 and **** p < 0.0001.


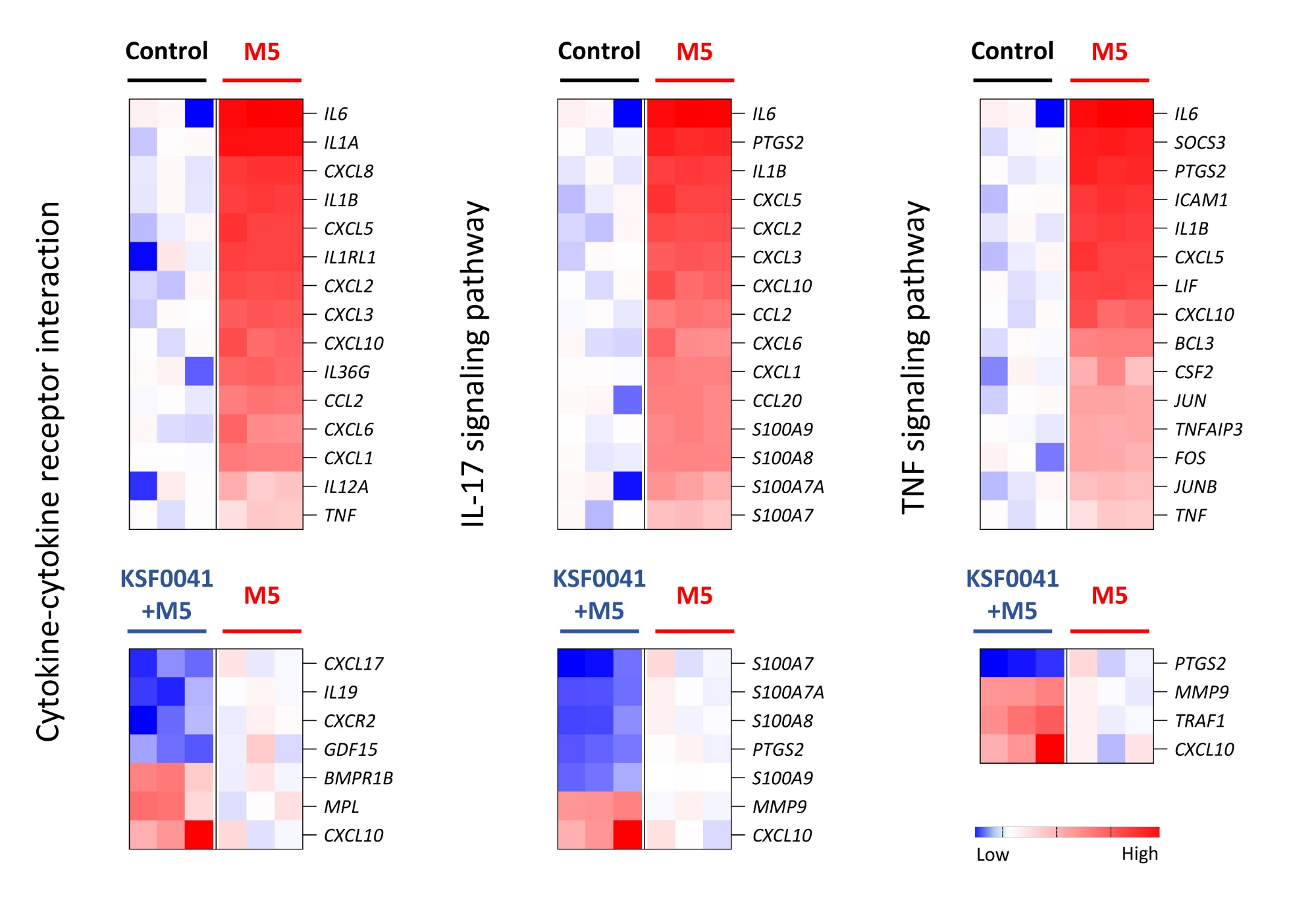


**Figure S3**. **The effects of M5 cytokines and polar microalgae extracts on the transcriptome of HaCaT cells.** Selected KEGG gene sets that were differentially expressed between unstimulated HaCaT cells (control) and M5 cytokine-stimulated HaCaT cells (upper) and between HaCaT cells pretreated with KSF0041 and stimulated with M5 cytokines and HaCaT cells only stimulated with M5 cytokines (lower). Heat map showing the log2 fold changes to the geometric mean of fragments per kilobase of exons per million fragments mapped + 0.01. The data were generated from a single experiment.

**
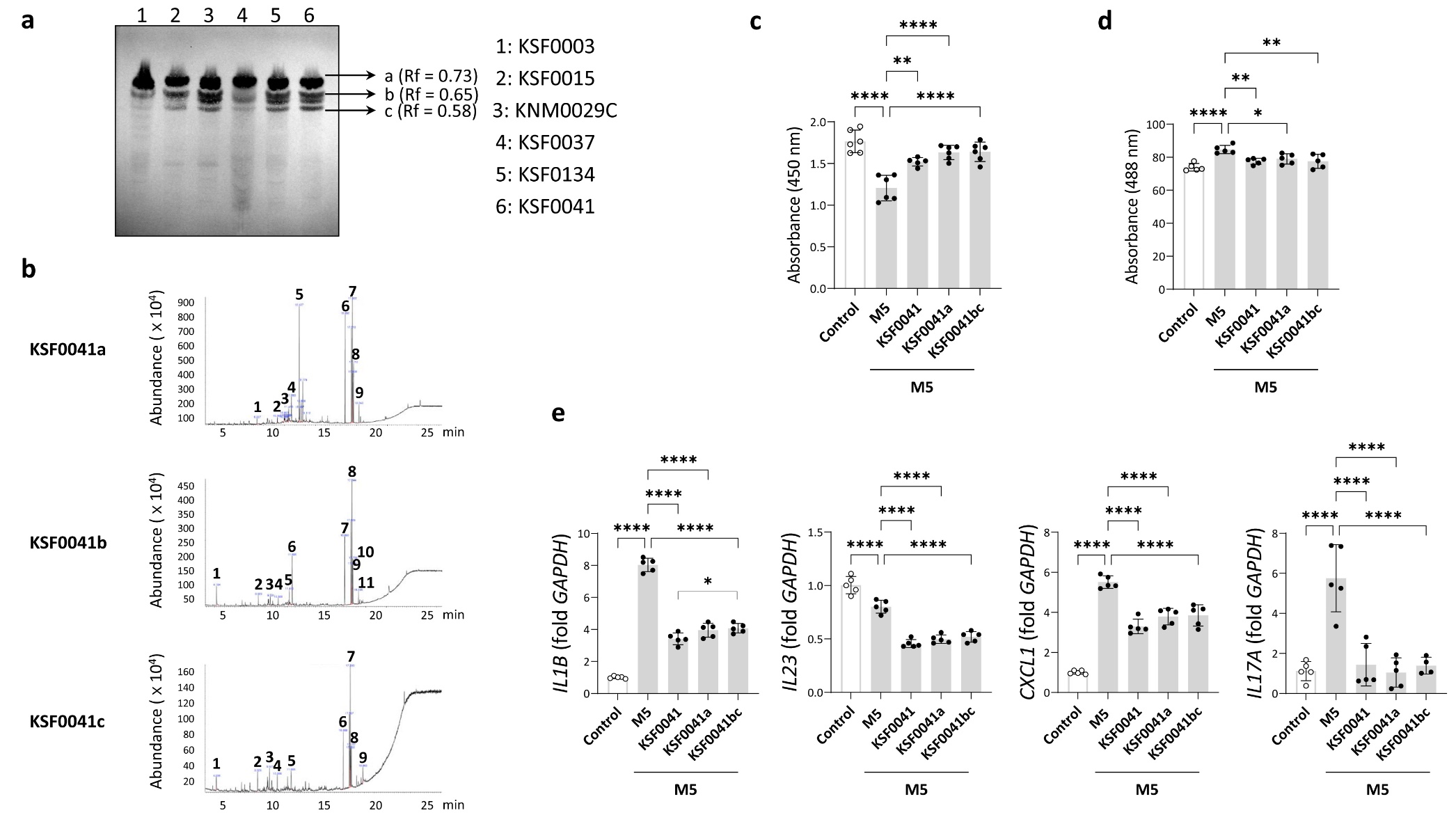
**

**Figure S4**. **Characteristics of the alkaloid fractions of polar microalgae extracts.** (a) The profile of alkaloid compounds in the polar microalgae extracts sprayed with Dragendorff’s reagent. Rf indicates the retention factor. (b) Chromatographic profiles of the alkaloid fractions of KSF0041 a, b, and c. (c) Viability of HaCaT cells that were pretreated overnight with the KSF0041 fractions and then treated with M5 cytokines and incubated for 18 h. (d) Fluorescence of 4-kDa fluorescein isothiocyanate-dextran fluxed from the apical to the basal compartment of transwells seeded with HaCaT cells treated with M5 cytokines after overnight incubation with the KSF0041 fractions. (e) mRNA expression of *IL1B*, *IL23*, *CXCL1*, and *IL17A* in HaCaT cells treated with M5 cytokines after an overnight incubation with the KSF0041 fractions; Data are presented as the mean ± SD. p-values are determined by one-way ANOVA with Tukey's multiple comparisons. * p < 0.05, ** p < 0.01, and **** p < 0.0001.

**3. Supplementary Table**

## Table S1. Primer sequences for real-time PCR.

| Target gene | Primer sequence |  |
| --- | --- | --- |
| *IL1B* | Forward: 5′- ATG ATG GCT TAT TAC AGT GGC AA -3′  Reverse: 5′- GTC GGA GAT TCG TAG CTG GA -3′ | |
| *CXCL1* | Forward: 5′- CAG TGT GTG GTC AAC ATT TCT CA -3′  Reverse: 5′- GCC CCT TTG TTC TAA GCC AG -3′ | |
| *IL23* | Forward: 5′- CTC AGG GAC AAC AGT CAG TTC -3′  Reverse: 5′- ACA GGG CTA TCA GGG AGC A -3′ | |
| *IL17A* | Forward: 5′- AGA TTA CTA CAA CCG ATC CAC CT -3′  Reverse: 5′- GGG GAC AGA GTT CAT GTG GTA -3′ | |
| *S100a8* | Forward: 5′- TTC CTT GCG ATG GTG ATA -3′  Reverse: 5′- ATG ATG ACT TTA TTC TGT AGA CA -3′ | |
| *S100a9* | Forward: 5′- TGA GGA GTG TAT GAT GCT GAT G -3′  Reverse: 5′- CAT TCC CTT TAG ACT TGG TTG G -3′ | |
| *Il1b* | Forward: 5′- GCA ACT GTT CCT GAA CTC AAC T -3′  Reverse: 5′- ATC TTT TGG GGT CCG TCA AC -3′ | |
| *Cxcl1* | Forward: 5′- AGT CAT AGC CAC ACT CAA GAA T -3′  Reverse: 5′- TCA GAA GCC AGC GTT CAC -3′ | |
| *Il17a* | Forward: 5′- GAC TTC CTC CAG AAT GTG AA -3′  Reverse: 5′- TGG AAC GGT TGA GGT AGT -3′ | |
| *GAPDH* | Forward: 5′- CTG GGC TAC ACT GAG CAC C -3′  Reverse: 5′- AAG TGG TCG TTG AGG GCA ATG -3′ | |
| *Gapdh* | Forward: 5′- CTG GTA TGA CAA TGA ATA CGG -3′  Reverse: 5′- GCA GCG AAC TTT ATT GAT GG -3′ | |

**Table S2. Compounds identified in KSF0041a extract.**

|  | Retention time (min) | Compound | % of total |
| --- | --- | --- | --- |
| 1 | 8.327 | Tetradecane | 0.782 |
| 2 | 10.306 | Hexadecane | 0.606 |
| 3 | 11.406 | 2,6-Diisopropylnaphthalene | 1.685 |
| 4 | 11.678 | Benzene, 1,1'-(1,2-cyclobutanediyl)bis-, trans | 4.227 |
| 5 | 12.422 | Neophytadiene | 14.635 |
| 6 | 16.885 | Cyclohexane, 1,3,5-triphenyl | 13.96 |
| 7 | 17.506 | Methadone N-oxide | 40.026 |
| 8 | 17.706 | 2-(2'-tolyl)-1H-indole | 7.64 |
| 9 | 18.243 | Ethyl 2-(2-phenethylbenzo[d][1,3]dioxol-2-yl)acetate | 2.772 |

**Table S3. Compounds identified in KSF0041b extract.**

|  | Retention time (min) | Compound | % of total |
| --- | --- | --- | --- |
| 1 | 4.193 | 1-Hexanol, 2-ethyl | 5.015 |
| 2 | 8.327 | Tetradecane | 1.975 |
| 3 | 9.537 | 2,4-Di-tert-butylphenol | 2.327 |
| 4 | 10.306 | Hexadecane | 1.193 |
| 5 | 11.406 | 2,4-Diphenyl-1-butene | 2.757 |
| 6 | 11.685 | Benzene, 1,1'-(1,2-cyclobutanediyl)bis-, trans | 10.389 |
| 7 | 16.885 | Cyclohexane, 1,3,5-triphenyl | 12.924 |
| 8 | 17.590 | Methadone N-oxide | 43.371 |
| 9 | 17.629 | 1H-Indole, 2-methyl-3-phenyl | 8.087 |
| 10 | 17.707 | 1H-Indole, 5-methyl-2-phenyl | 8.949 |
| 11 | 18.244 | Ethyl 2-(2-phenethylbenzo[d][1,3]dioxol-2-yl)acetate | 3.103 |

**Table S4. Compounds identified in KSF0041c extract.**

|  | Retention time (min) | Compound | % of total |
| --- | --- | --- | --- |
| 1 | 4.206 | 1-Hexanol, 2-ethyl | 3.891 |
| 2 | 8.327 | Tetradecane | 3.873 |
| 3 | 9.524 | 2,4-Di-tert-butylphenol | 5.364 |
| 4 | 10.306 | Hexadecane | 2.451 |
| 5 | 11.684 | [2.2]Paracyclophane | 6.448 |
| 6 | 16.885 | Cyclohexane, 1,3,5-triphenyl | 12.076 |
| 7 | 17.597 | Methadone N-oxide | 51.249 |
| 8 | 17.707 | 1H-Indole, 2-methyl-3-phenyl | 9.404 |
| 9 | 18.865 | 13-Docosenamide, (Z)- | 5.243 |

**References**

1. Akbarizare M, Ofoghi H, Hadizadeh M, Moazami N. In vitro assessment of the cytotoxic effects of secondary metabolites from Spirulina platensis on hepatocellular carcinoma. Egyptian Liver Journal. 2020;10:11.
